# Fuel Characteristics and Removal of AAEMs in Hydrochars Derived from Sewage Sludge and Corn Straw

**DOI:** 10.3390/molecules28020781

**Published:** 2023-01-12

**Authors:** Shuai Guo, Weinan Xiao, Zhaoyuan Liu, Deng Zhao, Kaixin Chen, Chenchen Zhao, Xingcan Li, Guangyu Li

**Affiliations:** 1 School of Energy and Power Engineering, Northeast Electric Power University, Jilin 132012, China; 2 Harbin Boiler Company Limited, Harbin 150046, China; 3 College of Vehicles and Energy, Yanshan University, Qinhuangdao 066000, China; 4 Northeast Electric Power Design Institute Co., Ltd. of China Power Engineering Consulting Group, Changchun 130000, China; 5 State Key Laboratory of High-Efficiency Utilization of Coal and Green Chemical Engineering, Ningxia University, Yinchuan 750021, China

**Keywords:** sewage sludge, corn straw, hydrothermal carbonization, dechlorination, dealkalization

## Abstract

Co-hydrothermal carbonization (Co-HTC) of sewage sludge (SS) and corn straw (CS) for fuel preparation is a waste treatment method that reduces the pre-treatment cost of solid waste and biomass fuel. Based on the response surface methodology (RSM), a test was designed to prepare SS and CS hydrochars using a hydrothermal high-pressure reactor. The test examined the higher heating value (HHV) and the concentrations of alkali metals and alkaline earth metals (AAEMs) and Cl. The HHV of SS-hydrochar decreased with an increase in reaction temperature, but that of CS-hydrochar increased. The yield of CS-hydrochar was at 26.74–61.26%, substantially lower than that of SS-hydrochar. Co-hydrochar has the advantages of HHV and an acceptable yield. The HHV of co-hydrochar was 9215.51–12,083.2 kJ/kg, representing an increase of 12.6–47.6% over single component hydrochar, while the yield of co-hydrochar was 41.46–72.81%. In addition, the stabilities of AAEM and Cl in the co-hydrochar were Mg > Ca > K > Na > Cl. SS and CS had a synergistic effect on dechlorination efficiency (DE), which had a negative effect on the removal efficiency (RE) of Ca and Na. The optimal hydrocharization conditions were a temperature of approximately 246.14 °C, a residence time of approximately 90 min, and a mixing ratio of SS–CS of approximately 57.18%. The results offer a way to utilize SS and CS by Co-HTC and convert them into low-chlorine and low-alkali fuel, thus pushing the improvement of this promising waste-to-energy technology.

## 1. Introduction

Recently, with the increase in urbanization in China, the output of sewage sludge (SS) has increased. The annual output of municipal sludge in China reached 60–90 million tons in 2020 [1]. SS is a heterogeneous mixture consisting of water, non-toxic organic carbon compounds, microbial pollutants (including pathogens), toxic organic and inorganic pollutants (such as heavy metals and polycyclic aromatic hydrocarbons), nitrogen, chlorine, phosphorus, and other inorganic compounds (e.g., silicates, aluminates, and compounds containing potassium, sodium, calcium, and magnesium). Incineration [2], absorption [3,4], composting, and landfilling [5] are traditional sludge disposal methods. Considering the increasing limitations of traditional treatment methods and the limitations of fossil fuel reserves, pyrolysis and hydrocharization (hydrothermal carbonization, HTC) are more effective than traditional treatment methods. (Co-)Pyrolysis [6,7] has attracted widespread attention because of the thorough sterilization of SS through high temperatures and the economic production of bio-oil, biogas, and biochar. However, the direct pyrolysis of SS requires a dehydration process. Because of the high moisture content (80%) of the sludge, direct pyrolysis leads to a decrease in the higher heating value (HHV) of the product and an increase in energy consumption. Compared with pyrolysis, HTC treatment of sludge is a more promising thermochemical treatment method. HTC can convert wet biomass into carbon-containing homogeneous hydrochar at a relatively low temperature (150–300 °C) and autogenous pressure. SS can be directly carbonized by HTC without dehydration. HTC has a higher yield than pyrolysis. However, because of the high ash content of the sludge produced by the HTC process, both volatile and carbon contents decrease, resulting in a decrease in the HHV. Furthermore, because of its high ash content and low heating value, hydrochar is not attractive for use as a solid fuel. Zheng et al. [8] conducted Co-HTC experiments on SS and kitchen waste. The results showed that, compared with the hydrochar of SS, the hydrochar derived from the Co-HTC of SS and kitchen waste had a higher C content and higher HHV. Thermogravimetric analysis (TGA) showed that the combustion behavior of co-hydrochar is better than that of SS-hydrochar. Lee et al. [9] prepared high-quality solid fuel for the Co-HTC process using sludge and microalgae. When the hydrothermal temperature was 210 °C, the HHV reached up to 19 MJ/kg, and the H/C and O/C ratios were 1.63 and 0.41, respectively. The fuel properties were comparable to those of lignite and bituminous coal. J.M. et al. [10] used TGA to study the thermal behavior of hydrochar from a Co-HTC of sawdust (SD) and sewage sludge (SS). HTC reduced the devolatilization performance of SS, while Co-HTC substantially improved the performance (7.38–23.69 times). Thermodynamic parameters indicated that the Co-HTC of SD and SS improved the pyrolysis reactivity of hydrochar derived from SD. Zhang et al. [11] found that sludge and pine sawdust had a considerable synergistic enhancement in terms of increased hydrochar yield along with organic matter and carbon retention. By combining SS and pine sawdust at a mass ratio of 1:1, a hydrochar yield of 58.11% ± 0.91% was obtained, with a high synergy coefficient.

The Co-HTC of SS and other substances can increase the potential of co-hydrochar as a solid fuel [12,13]. Currently, most scholars focus on the fuel characteristics of SS, and very few consider dechlorination and dealkalization. Zhang et al. [14] studied the Co-HTC of water hyacinth (WH) and polyvinyl chloride (PVC) based on response surface methodology. Statistical analysis showed that the reaction temperature was the key parameter that affected the dechlorination efficiency (DE), yield, HHV, energy recovery rate, and power consumption of hydrochar. The characterization results showed that hydrochar could harvest lower H/C and O/C ratios and provide excellent removal of inorganic substances. Huang et al. [15] proposed an innovative integrated process that used sawdust and PVC to produce clean solid biofuels. Using Co-HTC, the energy yield reached 74–81% and a substantial increase in the DE and removal efficiency (RE) of inorganic matter was noted. The results showed that the Co-HTC of PVC and biomass could effectively remove chlorine and inorganic substances through a positive synergistic effect. M.T. Reza. [16] studied the HTC of corn straw (CS), miscanthus, switchgrass, and rice husk. Owing to the removal of hemicellulose, the HTC treatment removed a high proportion of calcium, magnesium, sulfur, phosphorus, and potassium. HTC treatment reduced all heavy metals and increased the HHV. Yang et al. [17] selected CS samples as their research object. The effects of the process parameters on the physical and chemical properties of hydrochar and the removal of AAEMs were studied. The results showed that CS-hydrochar had a higher quality and energy yield than raw CS.

To date, many scholars have studied several fuel properties of the hydrochar of SS and biomass materials, including corn stalk, bamboo, wheat stalk, water hyacinth, microalgae, and peanut shells, and found that the hydrochar’s HHV, yield, energy yield, H/C, and O/C, etc., were improved. In addition, they found that SS with high moisture content can be directly carbonized hydrothermally without requiring dehydration. However, owing to the high ash content in SS, after HTC, both the volatile matter and carbon content decreased, resulting in a decrease in the HHV. The high ash content and low HHV of hydrochar are not conducive to its utilization as a solid fuel, its potential to become a solid fuel is low, and K, Na, Ca, Mg (AAEM), and Cl of SS cause slagging and corrosion of the equipment. As a common woody biomass agricultural waste, CS contains a certain amount of organic matter, mainly including proteins, sugars, and lipids. It has a higher HHV than SS, but it has poor hydrophilicity and low yield. The characteristics are not suitable for hydrochar alone. Besides, most of studies observed that biological raw materials can significantly improve either RE or DE of another substance in the co-hydrothermal process with other substances. However, few studies had focused on both DE and RE of AAEM during co-hydrothermal carbonization between SS and CS. 

In this study, co-hydrothermal carbonization of SS and CS was carried out via response surface methodology to achieve optimal dechlorination and dealkalization effects of SS and CS. The goals were as follows: (1) To study the effects of temperature, time, and mixing ratio on the performance of hydrothermal-carbon fuels, including solid yield and HHV; (2) to determine the removal rate of AAEM and Cl in the hydrochar and the influence of temperature, time, and mixing ratio on dechlorination and dealkalization; and (3) to explore the synergistic effect of SS and CS on the removal of AAEM and Cl in the Co-HTC process and optimize the co-hydrothermal process. This technology can reduce the pretreatment cost, improve the quality of the fuel, and realize the clean thermal conversion of SS and CS to the low-chlorine and low-alkali fuel.

## 2. Results and Discussion

### 2.1. Model Building

Analysis of variance was used to statistically evaluate the adequacy of the quadratic model. Based on the Box–Behnken design and experimental results, the quadratic regression model expressed the effects of variables on HHV, yield, RE of AAEM, and DE, respectively, from Equations (1)–(7):

(1) $$Y1=11453.12\;-\;311.93A+218.21B\;-\;6928.38C+364.11\mathrm{AB}\;-\;968.04\mathrm{AC}\;\;-\;8.75\mathrm{BC}\;-\;{35.30A}^{2}\;-\;624.13B^{2}+1439.34C^{2}$$

(2) Y2=0.58 − 0.13A − 0.015B+0.16C

(3) $$Y3=62.69+7.26A\;-\;2.86B+12.95C\;-\;1.49\mathrm{AB}\;-0.54\mathrm{AC}\;-1.79\mathrm{BC}+11.09A^{2\;}+8.27B^{2}\;-1.49C^{2}$$

(4) Y4=47.78+7.00A −1.85B+22.16C

(5) Y5=54.74+10.75A+2.75B+20.52C

(6) $$Y6=12.91\;-4.08A+5.08B+12.20C+7.10\mathrm{AB}+0.42\mathrm{AC}+12.11\mathrm{BC}+11.97A^{2}+28.98B^{2}\;-1.83C^{2}$$

(7) $$\;\;\;Y7=84.25+4.40A\;-0.79B\;-10.04C\;-2.62\mathrm{AB}+6.83\mathrm{AC}\;-1.07\mathrm{BC}\;-10.57A^{2}+8.98B^{2}\;-10.28C^{2}$$

where A, B, and C represent HTC conditions, which was the reaction temperature, residence time, and mixing ratio of SS to CS, respectively. Y1–Y7 are the estimated responses of HHV, yield, Ca, K, Mg, Na (RE), and DE, respectively. Supplementary Materials Table S1 shows the results of the ANOVA.

These analyses were performed on the response model given by ANOVA. The *p*-values were less than 0.05 for several models, indicating statistical significance. The lack of fit was minimal, indicating that the model is suitable for several responses. The quadratic polynomial regression models of the seven target quantities were all related to A, B, and C. Except for the yield, K, and Mg, the interaction terms of the quadratic polynomial regression models of the remaining target quantities, AB, AC, and BC, were significant, indicating that the response was interactive.

### 2.2. Response Surface/Contour Plots

#### 2.2.1. Response Surface Analysis of Fuel Characteristics

**Figure 1:**
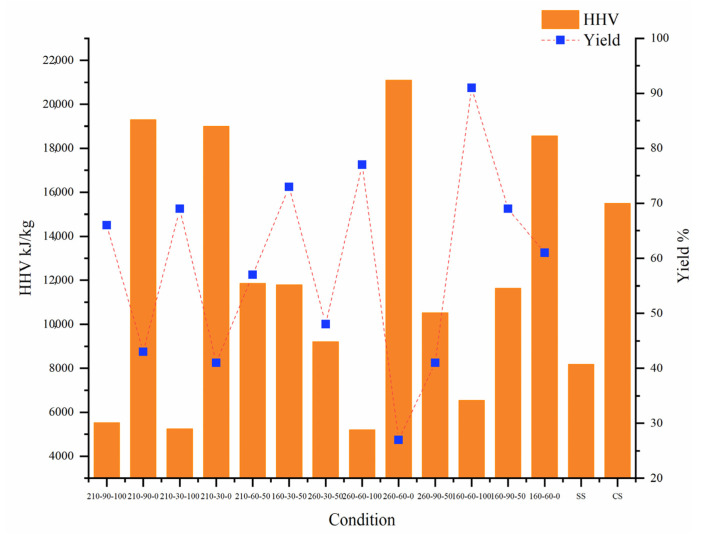
The HHV and yield of hydrochar and raw materials.

The HTC operating conditions play a vital role in determining the HHV of hydrochar. A response surface experiment design was used to study the HTC conditions. The HHV and yield of hydrochar and raw materials under different HTC conditions are shown in Figure 1. The HHV of raw CS was 15,500 kJ/kg, while that of raw SS was only 8185.8 kJ/kg. The HHV of SS-hydrochar decreased with increasing temperature and was lower for all SS-hydrochar tests than for raw SS. This is because, after HTC, SS-hydrochar has a lower carbon content and higher ash content than the raw materials [8]. As for CS, the HHV of CS-hydrochar increased with temperature and was higher than that of raw CS. CS contains large amounts of cellulose, hemicellulose, and lignin. The order of degradation for the same hydrothermal strength was as follows: lignin < cellulose < hemicellulose [18]. The HHV of lignin is greater than that of cellulose and hemicellulose, and the hydrochar generated under HTC conditions accumulated a large amount of lignin. The HHV of co-hydrochar was 9215.51–12,083.2 kJ/kg, representing an increase of 12.6–47.6% over single component hydrochar. 

**Figure 2:**
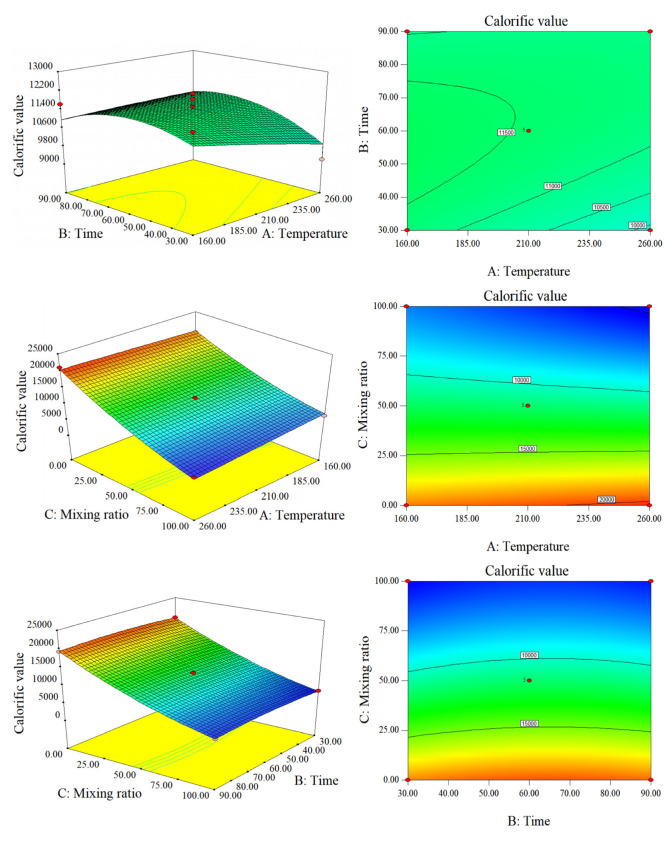
Response surface plots and contour plots of HHV.

Figure 2 shows that the influence of the blending ratio on HHV was considerably greater than that of temperature or time. This is primarily related to the large gap between the original CS and SS HHV. Temperature and time have an observable positive interaction on HHV, which is manifested by the prominent response surface, elliptical contour lines, and the curve and convexity of the contour lines. The yield of CS-hydrochar was very low at 26.74–61.26%, while the yield of co-hydrochar was 41.46–72.81%. The HHV reached a maximum when the blending ratio was 50%, the temperature was approximately 210 °C, and the residence time was approximately 60 min. Hemicellulose begins to hydrolyze at approximately 180 °C, cellulose begins to hydrolyze at approximately 210 °C, and lignin is not easily hydrolyzed within this temperature range [18,19]. It can also be seen from Figure 2 that the residence time had a negligible effect on the calorific value of SS-hydrochar, CS-hydrochar, and co-hydrochar at the same temperature (210 °C).

**Figure 3:**
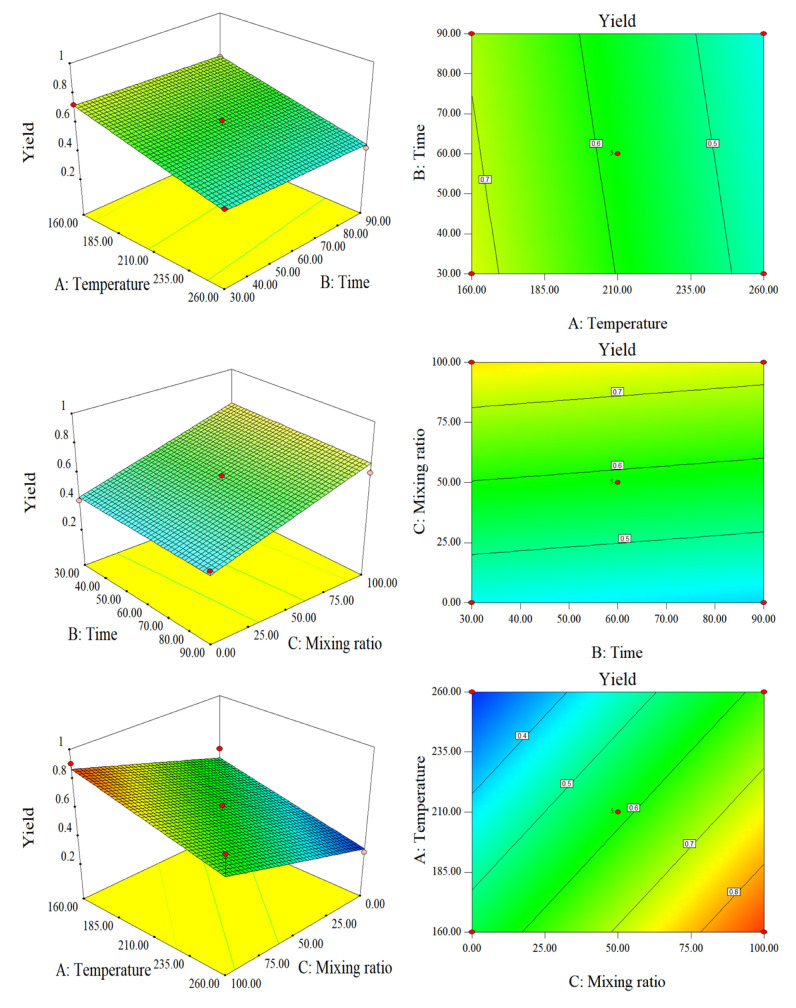
Response surface plots and contour plots of the yield.

The quality yield of hydrochar is also an important indicator of the amount of SS and CS converted into hydrochar in HTC. Figure 1 and Figure 3 show that, when the blending ratio was 50%, the quality yield decreased with increasing temperature. Time had minimal effect on the yield. This is because increasing the HTC operating conditions of SS will cause thermal decomposition and dissolution of SS macromolecules, resulting in a decrease in solid yield. As the reaction temperature increased from 210 °C to 260 °C, the quality yield of hydrochar decreased the most. According to related research, the substantial decomposition of hemicellulose and lignin in this temperature range are the primary causes for the high number of cellulose decomposition reactions (above 210 °C) [20]. SS contains many nitrogen compounds. During the hydrocharization process, soluble protein and carbohydrate hydrolysates were dissolved in the water phase. In addition, it is difficult for proteins to form hydrochar. It is also difficult for proteins to thermally decompose at approximately 250 °C to form hydrochar. Therefore, the higher the reaction temperature, the lower the yield. In addition, the mixing ratio had a negative impact on the yield. The HTC process degrades and reorganizes biomass polymers (proteins and polysaccharides in SS) into the liquid phase. As the temperature increased, the amount of soluble protein and carbohydrate hydrolysates in the water phase also increased, resulting in an increase in quality loss [21]. CS contains much more cellulose, hemicellulose, and lignin than SS. Under the same temperature and time of hydrothermal conditions, the yield was much lower than that of SS of the same quality. However, the yield trends of CS and SS were the same, owing to the hydrolysis of macromolecules, which decreased substantially with increasing temperature.

The addition of CS remarkably increased the HHV of the mixed hydrochar in the Co-HTC experiment, making it possible for these two waste materials to become fuels. However, this occurred at the expense of the yield.

#### 2.2.2. Response Surface Analysis of RE of AAEM and DE

##### Response Surface Analyses of RE of Na and K

**Figure 4:**
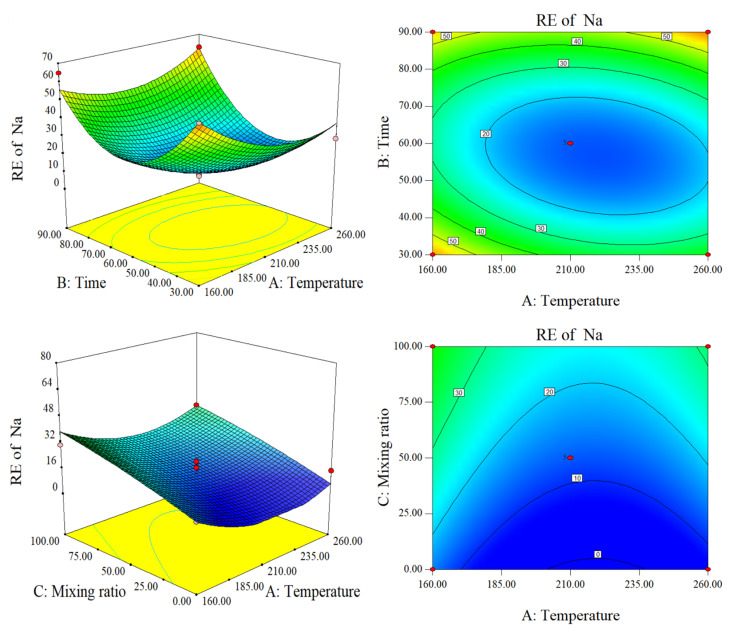

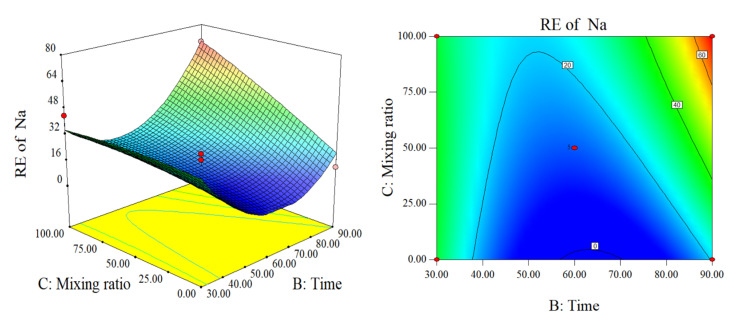
Response surface plots and contour plots of RE of Na.

Na and K are alkali metals. Supplementary Materials Table S2 shows that raw SS had almost the same Na and K content as raw CS. According to Figure 4, temperature and time have a very pronounced negative effect on the RE of Na. The RE contour lines on the Time versus Temperature map are elliptical in shape. Combining the response surface and the analysis of variance in Section 3.1, the mixing ratio had a substantially greater impact on the RE of Na than did temperature or time. When either time or temperature were held constant at 60 min or 210 °C, respectively, and the opposite parameter allowed to vary, RE increased with the mixing ratio. This may be related to the existence of bound water in SS, which is more suitable for hydrothermal treatment than CS. Combining the response surface and contour lines, AB and BC are significantly stronger than AC. The lowest points of each surface (lower RE) are blue areas near 210-60-50 (Temperature-time-mixing ratio). The mixing ratio remained unchanged at a temperature of 210 °C. As time increased, RE first decreased and then increased. The former is related to the stability of Na and its interaction with Cl. The latter is related to solubility, which will be discussed below.

**Figure 5:**
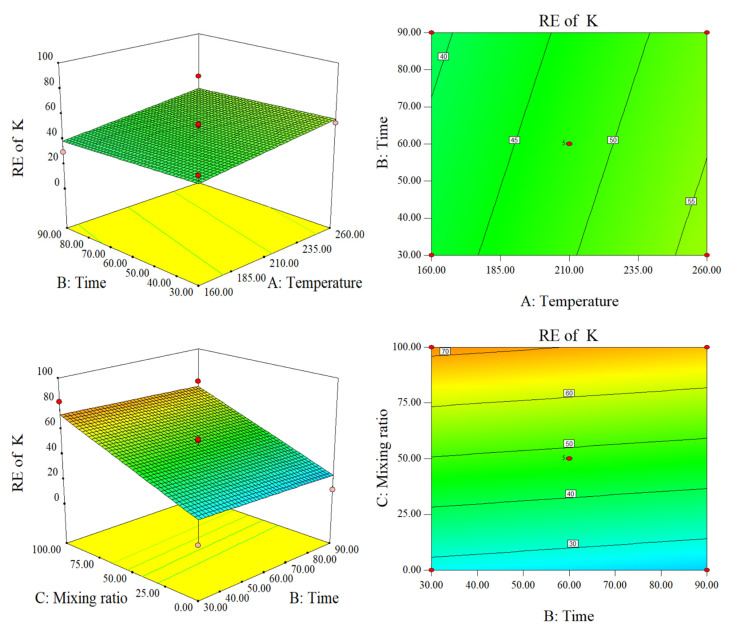

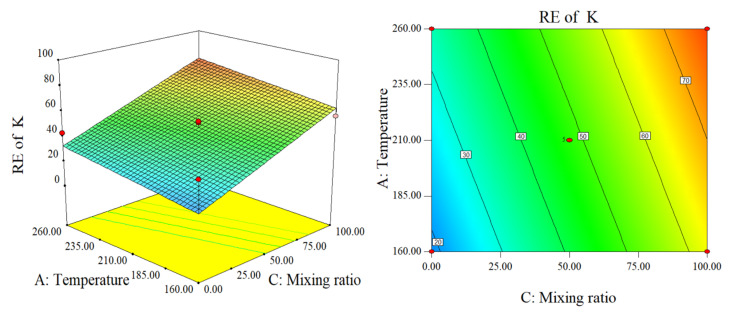
Response surface plots and contour plots of RE of K.

As shown in Figure 5, the effect of blending on RE (K) was far greater than that of temperature and time. RE increased with an increase in temperature and blending ratio. This is because RE (K) of SS and CS both increase with temperature, and RE has little relationship with time. The corresponding surface image was planar, and the contour lines were linear, indicating that there was no interaction. At equal temperatures, the RE of the SS was much greater than that of straw. This may be because lignin can fix K through physical adsorption and van der Waals forces. As lignin degrades, K is gradually released [22]. This phenomenon is related to the release of bound water from the SS in the HTC.

##### Response Surface Analysis of RE of Ca and Mg

**Figure 6:**
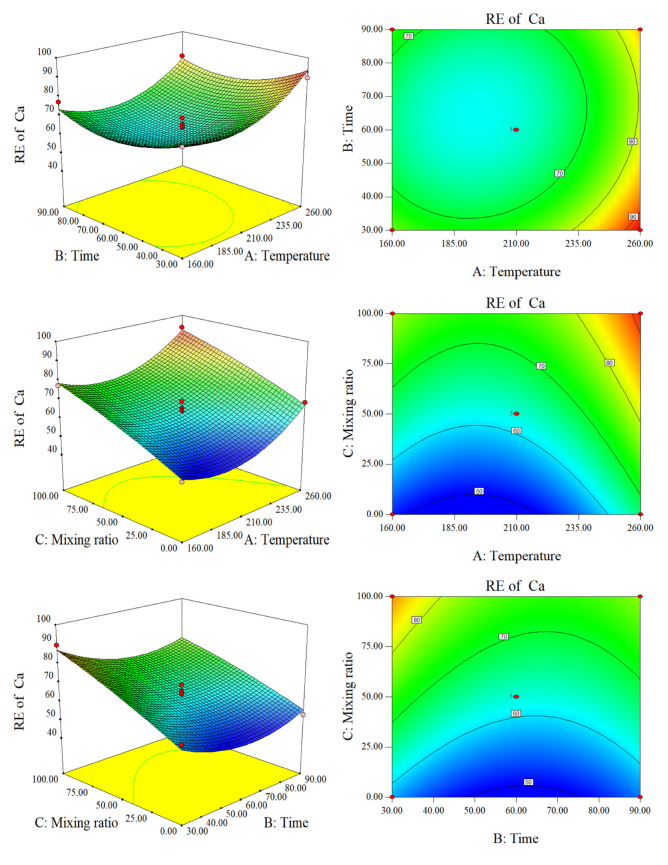
Response surface plots and contour plots of the RE of Ca.

Ca and Mg are both alkaline earth metals. As shown in Supplementary Materials Table S2, the concentrations of Ca and Mg in raw SS are much greater than those in raw CS. In addition, the effects of hydrothermal conditions on the RE are quite different. Figure 6 shows that there is a very pronounced negative interaction between the temperature and time of RE (Ca). This is also true of the temperature and the blending ratio. These three surface plots are concave and the contour graphs are elliptical. This may occur because the special network structure of lignin contains many active groups such as hydroxyl, carboxyl, and carbonyl, which can form complexes with Ca^2−^ [23]. When the residence time was 60 min and the control temperature was constant, the RE of the SS was greater than that of CS. This may be related to the slow adsorption and release characteristics of lignin. In addition, the Ca content in the original SS was much higher than that of CS, and the bound water of the SS was released during the HTC process. This can increase the ion exchange capacity of the hydrothermal system.

**Figure 7:**
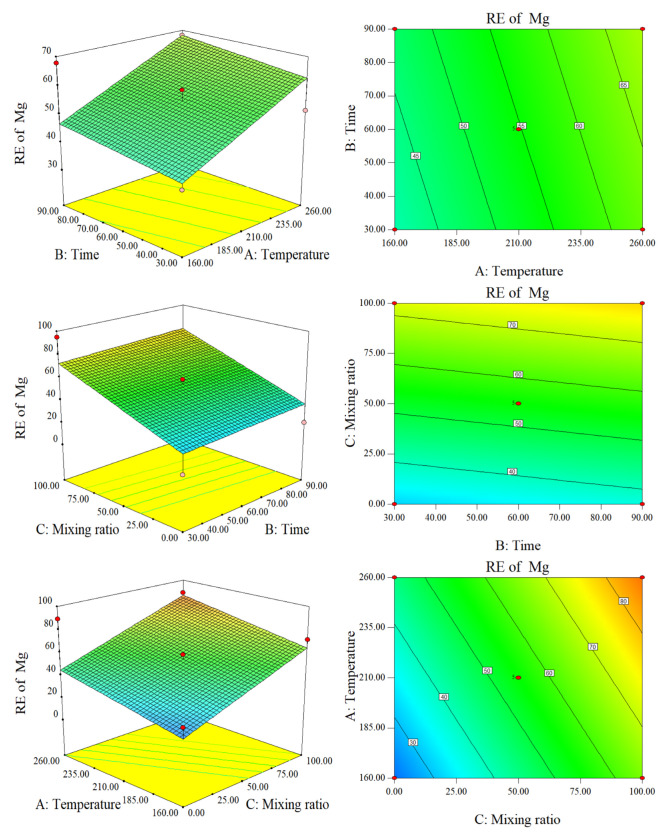
Response surface plots and contour plots of the RE of Mg.

According to Figure 7, RE (Mg) is positively correlated with temperature and time. As the temperature and time increased, the RE of the co-hydrochar increased substantially. According to the response surface diagram of temperature and blending ratio to RE, when the time was 60 min, the RE of SS-hydrochar was higher than that of CS at the same temperature. Time, temperature, and mixing ratio did not interact with RE. Compared with Ca, Mg may exist in hydrochar in a stable form. With an increase in temperature and time, Mg transformed from a stable state to a free state in the liquid phase.

##### Response Surface Analysis of DE

**Figure 8:**
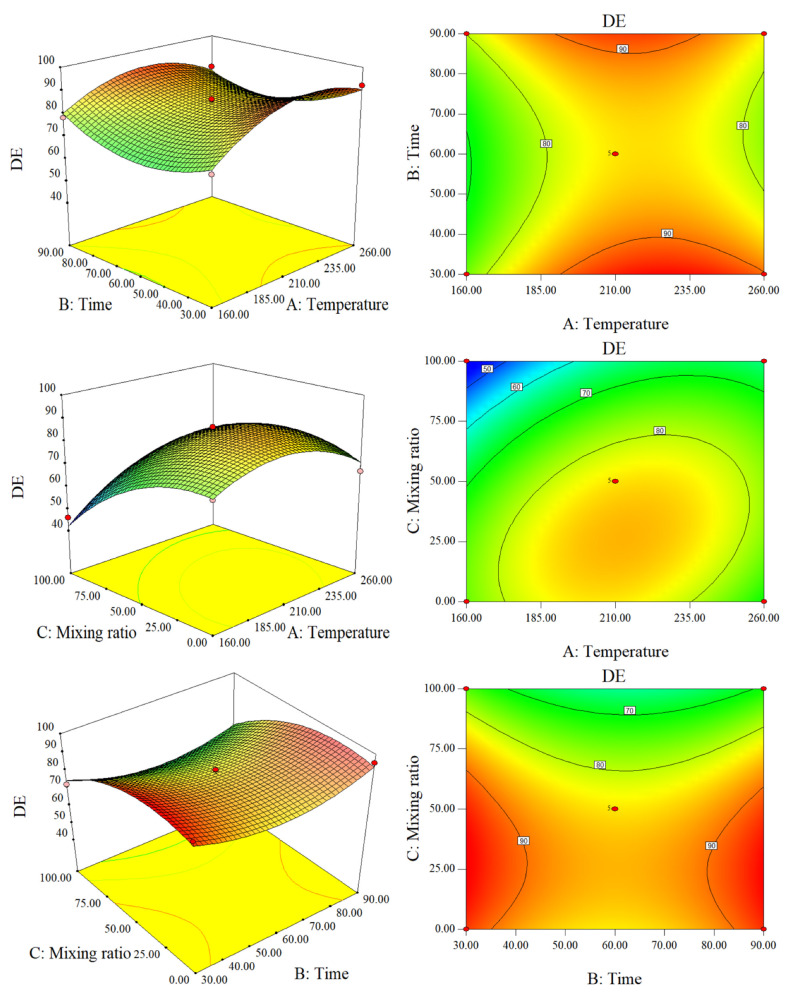
Response surface plots and contour plots of DE.

It can be seen from Figure 8 that, when the mixing ratio is 50% and the residence time is constant, the DE first increases and then decreases with an increase in temperature and the region where DE is at the maximum is roughly 185–235 °C. The effect of temperature on DE is far greater than the contribution of residence time when the temperature is lower than 185 °C. On the one hand, the increase in temperature strengthened the hydrolysis of cellulose and hemicellulose, thereby increasing free OH ions; on the other hand, it resulted in an increase in H ions and HCl in the autogenous pressure hydrothermal system. The OH ions released by the cellulosic biomass contributes to the release of Cl to the liquid phase, while the water and H ions and HCl further increase the hydrolysis of the cellulosic biomass; this synergistic effect may promote dehydration to increase chlorine efficiency [24]. In the range of 185–260 °C, the effect of residence time on DE cannot be ignored. With the increase in residence time, the DE shows a decreasing trend first and then an increasing one. This is because in this temperature range, the pressure of the hydrothermal system was large. When the residence time is short, Cl in the hydrochar is similar to the situation below 185 °C and DE is high. With the increase in residence time, the structure of the hydrochar changed and pores formed in the cracks and small protrusions on the surface of the hydrochar, which increased its adsorption capacity. These blocks could decompose to smaller ones and the entire hydrochar may lose much density, so that part of the Cl dissolved in the liquid phase may be re-adsorbed on the hydrochar [25]. As the residence time continued to increase, the energy continued to accumulate so that the Cl re-adsorbed on the hydrochar was released again. It can be observed from Figure 8 that the three-dimensional view of the response surface presents a very obvious protrusion, and the contour plot presents an obvious closed ellipse. There is a positive interaction between the effects of temperature and mixing ratio on DE. When the residence time was 60 min, the DE of CS hydrochar was much higher than that of SS hydrochar, but the difference in DE decreases with the increase in temperature. The DE of SS hydrochar is very close to that of pure CS hydrochar. This is because the Cl concentration in CS is several times higher than that of SS. After a suitable temperature for hydrolysis of cellulose and hemicellulose is achieved, the Cl is transferred to the aqueous phase. In addition, cellulose and hemicellulose were hydrolyzed in large quantities and the hydrolysis temperature of a small amount of lignin is much lower than that of macromolecules (protein, a small amount of lignin) in SS hydrochar. CS and SS have a synergistic effect on DE, and the highest DE obtained in this experiment was 95.48%. When the temperature was fixed at 210 °C and the mixing ratio at 75–100%, the residence time had almost no effect on the DE, which further increased with the decrease in the mixing ratio. When the mixing ratio was 0–75%, both the residence time and the mixing ratio affected the DE. When the mixing ratio remained unchanged, DE first decreases and then increases with the increase in residence time. The reason is probably that some of the Cl dissolved in the liquid phase may be re-adsorbed on the hydrochar, which is released again.

Under experimental conditions, AAEM and Cl in the gas phase were absent or negligible. Cl has the lowest stability and can be easily transferred to the liquid phase during HTC. Ca has high stability and is less likely to transfer into the liquid phase. According to the comparison of RE, Mg is more stable than Ca. The proportion of Mg in SS hydrochar and CS hydrochar is close and the hydrochar is relatively stable under different hydrothermal conditions. Comparing the RE of Na and K, it can be observed that the stability of Na is not as good as that of K, which might be because K is greatly affected by lignin. In sum, the order of stability of AAEM and Cl in HTC of SS and CS can be expressed as follows: Mg > Ca > K > Na > Cl.

### 2.3. Multi-Objective Optimization

Optimizing HTC operating conditions to recover hydrochar fuel is very important, especially from the perspective of process development. The ideal HTC process should obtain the maximum hydrochar yield and HHV (high fuel characteristics), RE of AAEM, and DE under lower reaction conditions. However, according to Section 2.2.2, the increase in HHV in this experiment was at the expense of yield. Combined with the actual factors, the limiting conditions are listed in Table 1.

Based on the analysis of fuel characteristics and the discussion of dechlorination and dealkalization mentioned above, combining HHV, yield, RE of AAEM, DE, and hydrothermal conditions, including the influence of temperature, time, mixing ratio, interaction, and the relationship between the mutual influence of indicators, the importance of the seven items were evaluated. The results are shown in Table 2.

Optimized by Design Expert software and the final prediction results in Table 3, the optimal condition was determined to be a temperature of approximately 246.14 °C, a residence time of approximately 90 min, and a blending ratio of approximately 57.18%. From Table 3, it can be observed that only the error of the yield is slightly large. Combined with 3.2.1, the improvement in the HHV of the co-hydrochar is at the expense of the yield, especially when the residence time is 90 min at 246 °C. The 246.14-90-57.18 was the optimal condition observed in this study in which the HHV and RE of AAEM and DE were excellent.

## 3. Materials and Methods

### 3.1. Materials

SS and CS were collected from Jilin City, Jilin Province. SS with an 80% moisture content was stored at 4 °C and used for the HTC test. Table 4 lists the primary properties of raw SS and CS. This technology avoided additional drying steps, and thus hydrous sludge was used. Part of the SS was dried, ground into a fine powder, and placed in a sealed plastic container for original sample analysis. CS was crushed using a crusher, sieved to less than 2.80 mm, and placed in a drying oven at 105 °C for 6 h.

### 3.2. HTC Experiments

HTC experiments of the target feedstocks (SS, CS, and their mixture) were conducted in a stirred batch reactor with an inner volume of 500 mL (stainless steel 316 L, GCF-type, Dalian Controlled Plant, Dalian, China). During each HTC run, a sample of SS (20 g, received base), CS (4 g, dry base), or their mixture dissolved in deionized water (200 mL) was loaded into the reactor vessel. The reactor was sealed during the experiment and the residual air was removed by nitrogen purging prior to heating to the specified temperature (160, 210, or 260 °C). An electric heater was used to maintain a constant temperature for 30, 60, or 90 min. The reaction pressure was the self-generated pressure range of 1.6 to 4.7 MPa. After the reaction, the solid-liquid mixture was collected and filtered into a filtrate and hydrochar. After drying at 105 °C for 24 h, the solid sample was ground to a fine powder and stored in a closed plastic pipe until analysis. Hydrocarbon samples are described as “A-B-C,” where A was the HTC temperature, B was the HTC residence time, and C was the mixing ratio of SS to CS. Indicators (yield, HHV, DE, and RE of AAEM) were selected as dependent variables, representing the response to changes in HTC conditions.

The yield was estimated using Equation (8), while the HHV was estimated using bomb calorimetry (SDAC6000, Hunan Sundy Science and Technology Co., Ltd., Zhuzhou, China). We added 5 mL of HNO_3_, 2 mL of HF, and 1 mL of HCl to 0.1 g of hydrochar for microwave digestion using a Jiangsu Tianrui Instrument Co., Ltd., Kunshan, China digestor. An ICP-MS2000 was used to analyze the AAEM in the digestion and collection solutions. The Cl concentration was obtained according to the “Determination of Halogen Content in Thermoplastic Elastomers-Oxygen Bomb Combustion-Ion Chromatography” (GB/T 34692-2017) method. Equations (9) and (10) were used to calculate RE and DE.

(8) Yield(%)=weight of dryhydrochar/weight of dry raw material

(9) RE=1 − 100% × X of hydrochar × Yield/Y of Solid Sample

(10) DE=1 − 100% × M of hydrochar × Yield/N of Solid Sample

AAEM and Cl were assumed to be absent in the gas phase in the range of 160–260 °C [26]. In Equations (9) and (10), X and M represent the concentration of the studied element in hydrochar according to different hydrothermal conditions. Variables Y and N represent the concentration of the studied element in SS and CS when analyzed separately or the arithmetic average of the two when mixed.

### 3.3. Experimental Design for Process Optimization

The response surface method was used to prove and predict the best working conditions according to the Box–Behnken design [27,28,29,30,31,32,33,34]. When many factors exist, the Box–Behnken design, as a three-level partial factor design, is a better choice than the central composite design [27,30,33]. The three factors were the hydrocharization temperature (160–260 °C), residence time (30–90 min), and mixing ratio (0–100%). The response values were hydrochar yield (%), HHV (kJ/kg), RE of AAEM (%), and DE (%). 17 tests were conducted for three factors at three levels, and the condition of 210-60-50 was repeated five times to prove the experimental reliability. According to the results of 17 tests, the best fitting model was calculated using Design Expert v.10 (Stat-Ease, Inc., Minneapolis, MN, USA) and the best working conditions were predicted and compared with the actual values. The initial parameters are listed in Supplementary Materials Table S3.

## 4. Conclusions

In this study, SS and CS were treated using the Co-HTC process. The fuel characteristics of co-hydrochar were improved, and the interaction between SS and CS was determined to affect the removal of Cl and AAEM in hydrochar. 

The HHV of co-hydrochar was 12.6–47.6% higher than that of SS-hydrochar, and the yield was also higher than that of CS-hydrochar. The addition of straw improved the HHV of the SS, and the addition of SS compensated for the low yield of CS. SS and CS had a synergistic effect on DE and a certain negative effect on the RE of Ca and Na. The order of stability of AAEM and Cl in the hydrothermal system of SS and CS was Mg > Ca > K > Na > Cl. In the Co-HTC process, the temperature and time had significant effects on the RE of Na, while the effect on K is negligible; there is a very obvious interaction between temperature, time, and mixing ratio on the RE of Ca, which was very little in case of the RE of Mg; the synergistic effect of SS and CS promotes the increase in DE. Finally, considering the maximum yield and HHV, RE of AAEM, and DE of hydrochar, the optimal hydrocharization condition was a temperature of approximately 246.14 °C, a residence time of approximately 90 min, and a mixing ratio of SS and CS of approximately 57.18%.

In this study, only three key factors, temperature, time, and mixing ratio, were considered in the optimization process, and the influence of water content and particle size was not considered. Furthermore, this study only considers DE and RE to evaluate slagging and corrosion. In the future, more indicators should be studied to further evaluate the characteristics of hydrochar, and cost analysis and environmental estimations of this technology should also be further studied.

## Figures and Tables

**Table 1:** Variable constraint table.

Name	Goal	Lower	Upper	Lower	Upper
		Limit	Limit	Weight	Weight
A: Temperature	is in range	160	260	1	1
B: Time	is in range	30	90	1	1
C: Mixing ratio	is in range	0	100	1	1
HHV	is in range	10,000	21,102.4	1	1
Yield	is in range	50	90.6	1	1
Ca (RE)	maximize	50.31	93.18	1	1
K (RE)	maximize	8.82	81.91	1	1
Mg (RE)	maximize	14.52	95.03	1	1
Na (RE)	maximize	7.5	68.92	1	1
Cl (DE)	maximize	46.06	95.48	1	1

**Table 2:** Evaluation of the importance of target variables.

Name	Goal Importance (1–5)
HHV (kJ/kg)	3
Yield (%)	3
K (RE)	2
Na (RE)	5
Ca (RE)	4
Mg (RE)	2
Cl (DE)	5

**Table 3:** Optimization and experimental results and error analysis.

.	Condition	HHV	Yield	K (RE)	Ca (RE)	Na (RE)	Mg (RE)	Cl (DE)	Desirability
Optimization results	246.14-90-57.18	10,000	50.00	79.6	54.2	68.2	58.9	87.1	0.74
Experimental results	246.14-90-57.18	9193	40.1	73.8	58.3	69.1	55.7	99.6	
Error		8.8%	24.7%	7.8%	7.2%	1.3%	5.7%	12.6%	

**Table 4:** Main characteristics of the employed SS and CS.

Sample	Proximate Analysis (%, Air Dry)				HHV (kJ/kg)	Ultimate Analysis (%, Air Dry)				
	M	A	V	FC		C	H	O	N	S
SS	3.84	58.80	32.66	4.70	8200	18.03	2.85	13.81	2.07	0.60
CS	4.81	5.41	76.56	13.22	17,820	48.73	6.65	33.20	0.92	0.28

## Data Availability

Not applicable.

## Supplementary Materials

The following supporting information can be downloaded at: https://www.mdpi.com/article/10.3390/molecules28020781/s1, Table S1: Analysis of variance; Table S2: The contents of AAEM and Cl; Table S3: Three-factor, three-level experimental design and results.

## Sample Availability

Samples of the compounds are available from the authors.
