# Supplementary material for: Fuel Characteristics and Removal of AAEMs in Hydrochars Derived from Sewage Sludge and Corn Straw
Source: Molecules. 2023 Jan 12;28(2):781. doi: 10.3390/molecules28020781 (PMC9862732; doi:10.3390/molecules28020781)
Supplement: Supplementary file 1 [file molecules-28-00781-s001.zip › molecules-2155028-supplementary.pdf]

Supplementary Data

Table S1 shows the analysis of variance of various target quantities in the experiments. This paper focuses on the significance and lack of fit of the model.

Table S1. Analysis of variance.

Source	SS	df	MS	*F*-Value	*p*-Value	
HHV						
Model	4.00E+08	9	4.44E+07	66.97	< 0.0001	significant
A	7.78E+05	1	7.78E+05	1.17	0.3144	
B	3.81E+05	1	3.81E+05	0.57	0.4731	
C	3.84E+08	1	3.84E+08	579.41	< 0.0001	
AB	5.30E+05	1	5.30E+05	8.00E-01	0.4008	
AC	3.75E+06	1	3.75E+06	5.66	0.049	
BC	3.06E+02	1	3.06E+02	4.62E-04	0.9835	
A^2	5.25E+03	1	5.25E+03	7.92E-03	0.9316	
B^2	1.64E+06	1	1.64E+06	2.47	0.1597	
C^2	8.72E+06	1	8.72E+06	13.16	0.0084	
Residual	4.64E+06	7	6.63E+05			
Lack of fit	3.05E+06	3	1.02E+06	2.56	0.1927	not significant
Pure error	1.59E+06	4	3.97E+05			
total	4.04E+08	16				
Yield						
Model	0.34	3	0.11	31.9	< 0.0001	significant
A	0.13	1	0.13	35.2	< 0.0001	
B	1.90E-03	1	1.90E-03	0.53	0.4779	
C	0.21	1	0.21	59.97	< 0.0001	
Residual	0.046	13	3.56E-03			
Lack of Fit	0.04	9	4.44E-03	2.81	0.1663	not significant
Pure Error	6.32E-03	4	1.58E-03			
Cor Total	0.39	16				
K						
Model	4346.89	3	1448.96	10.23	0.001	significant
A	391.72	1	391.72	2.77	0.1203	
B	27.53	1	27.53	0.19	0.6666	
C	3927.64	1	3927.64	27.72	0.0002	
Residual	1841.7	13	141.67			
Lack of Fit	1673.62	9	185.96	4.43	0.0829	not significant
Pure Error	168.08	4	42.02			
Cor Total	6188.59	16				
Na						
Model	6624.44	9	736.05	8.97	0.0043	significant
A	132.93	1	132.93	1.62	0.2437	
B	206.65	1	206.65	2.52	0.1565	
C	1190.96	1	1190.96	14.51	0.0066	
AB	201.64	1	201.64	2.46	0.161	
AC	0.7	1	0.7	8.50E-03	0.9291	
BC	586.12	1	586.12	7.14	0.0319	
A^2	603.31	1	603.31	7.35	0.0301	

B^2	3535.62	1	3535.62	43.09	0.0003	
C^2	14.1	1	14.1	0.17	0.6909	
Residual	574.36	7	82.05			
Lack of Fit	451.72	3	150.57	4.91	0.0791	not significant
Pure Error	122.64	4	30.66			
Cor Total	7198.8	16				
Ca						
Model	2700.47	9	300.05	12.29	0.0016	significant
A	421.23	1	421.23	17.25	0.0043	
B	65.61	1	65.61	2.69	0.1452	
C	1341.62	1	1341.62	54.95	0.0001	
AB	8.85	1	8.85	0.36	0.5661	
AC	1.19	1	1.19	0.049	0.8317	
BC	12.89	1	12.89	0.53	0.4911	
A^2	517.4	1	517.4	21.19	0.0025	
B^2	287.99	1	287.99	11.79	0.0109	
C^2	9.31	1	9.31	0.38	0.5564	
Residual	170.92	7	24.42			
Lack of Fit	65.06	3	21.69	0.82	0.547	
Pure Error	105.86	4	26.46			
Cor Total	2871.39	16				
Mg						
Model	4352.96	3	1450.99	3.94	0.0335	significant
A	924.5	1	924.5	2.51	0.1371	
B	60.72	1	60.72	0.16	0.6913	
C	3367.74	1	3367.74	9.14	0.0098	
Residual	4787.44	13	368.26			
Lack of Fit	4355.88	9	483.99	4.49	0.0811	not significant
Pure Error	431.56	4	107.89			
Cor Total	9140.4	16				
Cl						
Model	2411.09	9	267.9	24.47	0.0002	significant
A	154.88	1	154.88	14.15	0.0071	
B	4.95	1	4.95	0.45	0.5231	
C	807.02	1	807.02	73.71	< 0.0001	
AB	27.35	1	27.35	2.5	0.158	
AC	186.6	1	186.6	17.04	0.0044	
BC	4.6	1	4.6	0.42	0.5375	
A^2	470.84	1	470.84	43	0.0003	
B^2	339.75	1	339.75	31.03	0.0008	
C^2	444.72	1	444.72	40.62	0.0004	
Residual	76.64	7	10.95			
Lack of Fit	62.59	3	20.86	5.94	0.0591	not significant
Pure Error	14.06	4	3.51			
Cor Total	2487.73	16				

The RE of AAEM and DE in this paper are calculated by Equations (9) and (10) from the corresponding content and yield. The contents of AAEM and Cl in each working condition are shown in Table S2.

Table S2. The contents of AAEM and Cl.

Condition	Ca(mg/kg)	K(mg/kg)	Mg(mg/kg)	Na(mg/kg)	Cl(mg/kg)
160-30-50	19129.0	5530.5	2689.2	4866.4	375
160-60-0	3717.8	4717.8	891.9	1240.8	609
160-60-100	19740.1	6444.6	4922.9	2503.5	280
160-90-50	33980.0	3718.6	7542.0	5554.2	314
210-30-0	4274.4	919.5	380.9	2760.3	160
210-30-100	39456.2	10142.5	10563.6	3763.1	142
210-60-50	16341.5	4627.6	7173.8	1358.3	226
210-60-50	16722.2	5851.6	5954.7	667.2	240
210-60-50	17606.3	5971.8	9251.7	974.7	342
210-60-50	14271.0	4703.1	4973.5	1669.7	284
210-60-50	15158.6	4405.6	6134.5	614.0	259
210-90-0	3758.3	1241.9	531.8	937.2	160
210-90-100	33101.6	8940.1	7318.6	5910.2	152
260-30-50	22958.7	6091.8	3534.7	2352.7	167
260-60-0	4863.7	4510.5	2343.2	1143.9	3080
260-60-100	40999.2	9185.9	9825.4	2712.5	166
260-90-50	22041.8	7259.4	4686.5	5112.5	264
CS	7108.5	10422.7	2624.1	7794.5	1520
SS	43999.2	12381.9	11116.0	8574.8	411

The experimental conditions and results are shown in Table S3

Table S3. Three-factor, three-level experimental design and results.

Run	1	2	3	4	5	6	7	8	9	10	11	12	13	14	15	16	17
Temperature (℃)	210	160	210	210	160	260	210	210	160	210	210	260	210	260	260	160	210
Time (min)	60	90	60	90	30	60	30	60	60	30	90	90	60	60	30	60	60
Mixing ratio (%)	50	50	50	0	50	0	0	50	0	100	100	50	50	100	50	100	50
HHV（kJ/kg）	11529.6	11643.7	11349.3	19301.6	11790.3	21102.4	18992.9	11856.3	18567.5	5252.53	5526.23	10525.4	10447.2	5210.7	9215.51	6548	12083.2
Yield (%)	58.21	68.60	56.04	43.17	72.81	26.74	41.14	50.58	61.26	69.47	66.33	41.46	57.19	76.54	48.47	90.65	61.48
Ca:RE (%)	63.95	77.23	65.44	52.87	74.86	68.42	60.13	68.9	50.31	89.67	75.23	86.26	55.85	93.18	89.84	77.25	59.32
K:RE (%)	40.59	30.03	51.32	11.92	48.5	43.28	8.82	52.37	43.54	81.91	72.2	63.67	41.25	74.19	53.43	56.52	38.64
Mg:RE (%)	39.22	67.85	51.43	20.27	39.14	89.29	14.52	31.88	32.7	95.03	65.84	68.22	58.6	88.39	51.45	71.66	45.1
Na:RE (%)	16.6	64.77	8.15	12.02	59.46	14.68	35.41	11.91	15.31	43.89	68.92	62.46	20.4	31.63	28.75	30.59	7.5
Cl:DE (%)	86.37	78.31	86.07	95.48	74.69	67.08	94.87	82.1	75.46	72.58	68.9	85.4	83.21	65	92.24	46.06	83.51
